# Computational pathology model to assess acute and chronic transformations of the tubulointerstitial compartment in renal allograft biopsies

**DOI:** 10.1038/s41598-024-55936-3

**Published:** 2024-03-04

**Authors:** Renaldas Augulis, Allan Rasmusson, Aida Laurinaviciene, Kuang-Yu Jen, Arvydas Laurinavicius

**Affiliations:** 1 Department of Pathology and Forensic Medicine, Institute of Biomedical Sciences of the Faculty of Medicine, Vilnius University, M. K. Ciurlionio Str. 21, 03101 Vilnius, Lithuania; 2 National Centre of Pathology, Vilnius University Hospital Santaros Klinikos, P. Baublio Str. 5, 08406 Vilnius, Lithuania; 3 Department of Pathology and Laboratory Medicine, University of California, Davis School of Medicine, Sacramento, CA USA

**Keywords:** Digital pathology, Digital image analysis, Machine learning, Deep learning, Morphometry, Multivariate analysis, Kidney, Nephropathology, Nephrology, Kidney, Computational models, Image processing

## Abstract

Managing patients with kidney allografts largely depends on biopsy diagnosis which is based on semiquantitative assessments of rejection features and extent of acute and chronic changes within the renal parenchyma. Current methods lack reproducibility while digital image data-driven computational models enable comprehensive and quantitative assays. In this study we aimed to develop a computational method for automated assessment of histopathology transformations within the tubulointerstitial compartment of the renal cortex. Whole slide images of modified Picrosirius red-stained biopsy slides were used for the training (n = 852) and both internal (n = 172) and external (n = 94) tests datasets. The pipeline utilizes deep learning segmentations of renal tubules, interstitium, and peritubular capillaries from which morphometry features were extracted. Seven indicators were selected for exploring the intrinsic spatial interactions within the tubulointerstitial compartment. A principal component analysis revealed two independent factors which can be interpreted as representing chronic and acute tubulointerstitial injury. A K-means clustering classified biopsies according to potential phenotypes of combined acute and chronic transformations of various degrees. We conclude that multivariate analyses of tubulointerstitial morphometry transformations enable extraction of and quantification of acute and chronic components of injury. The method is developed for renal allograft biopsies; however, the principle can be applied more broadly for kidney pathology assessment.

## Introduction

Kidneys are the most frequently transplanted organs, with a total of around 92 thousand transplants performed worldwide in 2021^1^. Furthermore, this number represents an increase by over 14% since 2020, but a shortage of donor kidneys lead to an overall 70 thousand deaths^2^. Today, managing patients with kidney allografts is largely based on biopsy diagnosis^3–5^. To standardize kidney transplant diagnoses, the Banff classification system was proposed in 1991 and has since been updated periodically^6^. This continuous evolution of the Banff system is necessary for incorporating new scientific evidence to improve patient care and clinical practices. On the other hand, it necessitates ongoing education and adoption among pathologists and clinicians, which introduce more complexity that leads to challenges in consistent practical application and reproducibility^7,8^. Overall, the evaluation of transplant biopsies remains semiquantitative and subjective, and is further confounded by variability between observers^9^. Therefore, robust tools and methodologies for accurate, reproducible and quantitative assessment of biopsies are in great demand.

Advances in digital pathology, enabled by whole slide imaging (WSI) technologies with high-capacity digital image analysis (DIA) and deep learning (DL) methods, open new horizons for computer-aided diagnosis in many domains of tumor and non-tumor pathology^10,11^. DL-based methods enable automatic segmentation and classification of tissue compartments and tumor tissue detection^12^, classification of tumor histology patterns^13^, prediction of molecular tumor profiles as well as patient survival from H&E-stained tissue slides^14^.

Application of computational methods in the field of nephropathology has demonstrated great potential for studying the rather intricate renal histomorphology^15–19^. Firstly, DL-based methods enable robust segmentation and classification of kidney compartments, for example, segmenting glomeruli^20,21^. In particular, Jayapandian et al. reported DL-based segmentation of renal glomeruli, tubules, arteries, peritubular capillaries, and other structures across multiple stains and pathology laboratories^22^. Their study was based on 125 biopsies with minimal change disease from the patients with nephrotic syndrome which were mostly represented with minor histology changes. Bouteldja et al.^23^ presented a DL algorithm for accurate multiclass segmentation of PAS WSI. Their algorithm was trained using a large number of expert-based annotations and showed high performance in tasks like histology segmentation. Secondly, DL algorithms can be trained to recognize histology patterns specified by pathologists: Ginley et al.^24^ reported an automated computational detection of interstitial fibrosis and tubular atrophy (IFTA) along with glomerulosclerosis; their analysis outputs (IFTA % calculated by dividing total area of IFTA by the total area of the cortex) correlated with pathologists’ assessment and patient-outcome variables. Thirdly, DL can provide end-to-end solutions to predict clinical outcomes: Kers et al.^25^ used 5,844 WSI of kidney allograft biopsies from 1948 patients to pre-classify histology of kidney allograft biopsies into three main broad categories (normal, rejection, and other diseases).

Before the availability of DL, many studies applied DIA to assess renal histomorphology. Commonly, measurement of IFTA, glomerulosclerosis, and immune deposits were sought from histochemistry and immunohistochemistry (IHC) stains. For example, Dao et al.^26^ assessed interstitial fibrosis in Picrosirius red images of kidney allografts, Hunter et al.^27^ constructed a set of morphometry indicators from Picrosirius red images with addition of polarized light, Brazdziute et al.^28^ measured C4d deposition in peritubular capillaries.

In 2016, Farris et al.^29^ used trichrome, periodic acid-Schiff (PAS), and collagen III IHC to extract a broad set of morphometry indicators in search of their correlates between renal cortex and medulla; they applied multivariate statistics to cluster the features and assess their informative value. This approach is important since the morphometry features of renal histology objects are often interdependent. Consequently, the morphometric transformations in acute and chronic renal pathology processes may be impacted by specific contributions of the pathologies, while some individual metrics maybe redundant or biased. For example, IFTA may be represented by varying degrees of interstitial expansion due to fibrosis and/or tubular area shrinkage due to atrophy in the inflammatory or ischemic process. Furthermore, acute tubular injury and/or interstitial inflammation can develop in the context of IFTA which can obscure the quantification of the processes. Recently, Vaulet et al.^30^ demonstrated the potential of cluster analyses to extract chronic allograft phenotypes from pathology Banff scores; they identified four ”chronic clusters” associated with graft outcome which were independent of the acute lesions, but partially overlapped with the existing Banff IFTA classification categories. In other words, the clustering revealed phenotypes which could provide a better representation of chronic renal injury pathways by taking into account intrinsic interactions within the Banff score dataset. Multivariate analyses based on continuous data obtained by DIA of renal tissue could further increase the precision of computational models to assess acute and chronic components of injury.

The DL-based and DIA-based morphometry models can be synergized since high-capacity segmentation of the renal tissue compartments can serve as a sampling step for subsequent quantification of histopathology features. Recently, Holscher et al.^31^, presented a computational framework based on DL for semantic segmentation of glomeruli, tubules, and arteries with subsequent large-scale extraction of interpretable histomorphometry features; in particular, they identified a set of tubular and glomerular metrics associated with progression of renal disease that could supplement current prognostic models in IgA nephropathy.

The tubulointerstitial compartment is involved in both acute and chronic renal injury, in both native and transplanted kidneys. Therefore, we aimed to develop a computational model utilizing DL-based segmentation of renal cortex and its components, with subsequent extraction of morphometry features and multivariate analyses of the interpretable and continuous data set to independently represent acute and chronic transformations within the tubulointerstitial compartment.

## Results

### Data characteristics

**Figure 1:**
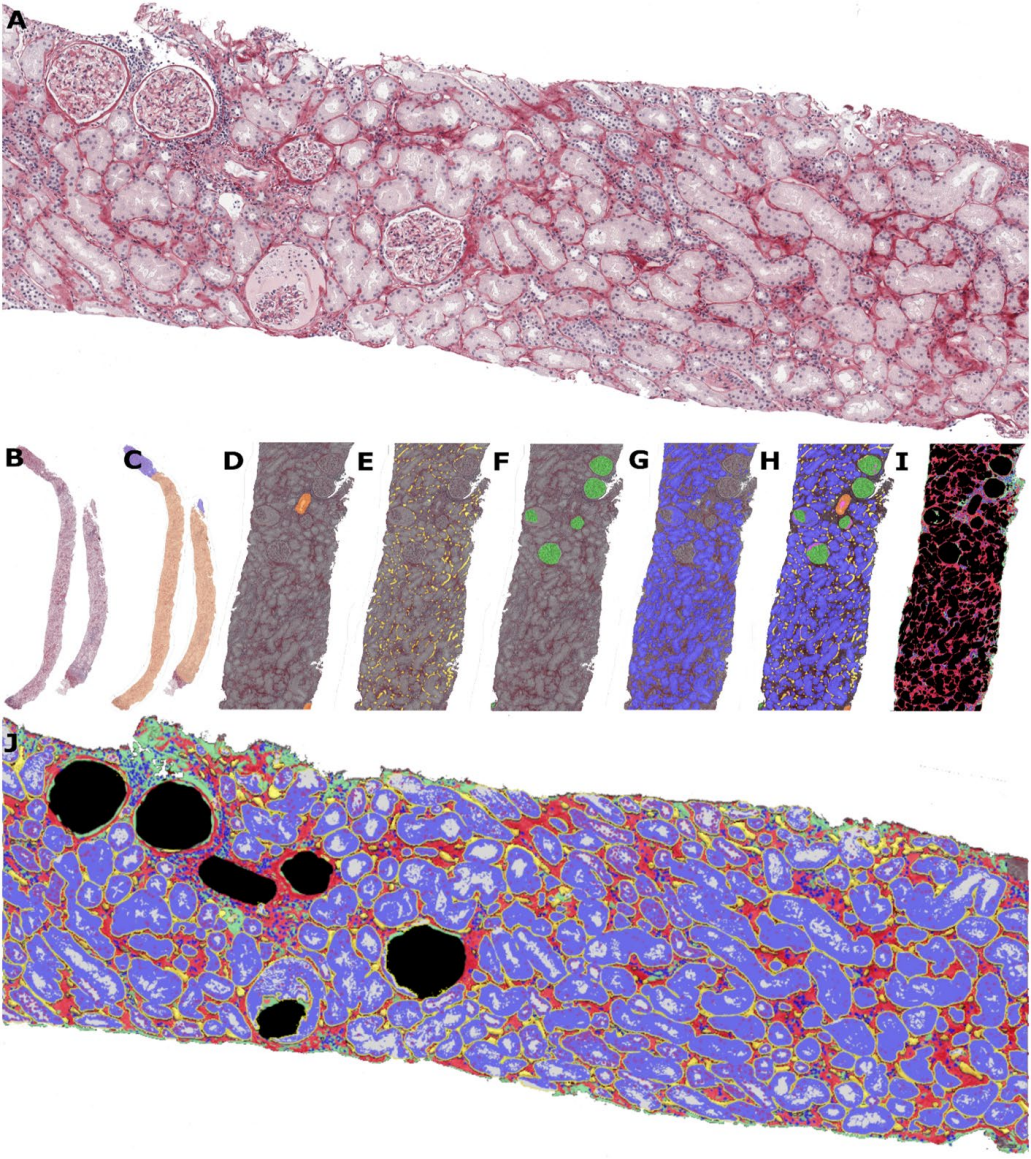
Image analysis workflow and output examples. (**A**): Modified Picrosirius red staining: red collagen, light yellow cell cytoplasm, and blue cell nuclei. (**B**): Whole slide image. (**C**–**G**): Classified renal structures, respectively cortex (**C**, light orange) and medulla (**C**, light blue); veins and arteries (**D**, orange); capillaries (**E**, yellow); glomeruli (**F**, green); tubules (**G**, blue). (**H**): Classified renal independent renal structures assembly and Overlap QA. (**I**): Residual tissue extraction. (**J**): Area quantification if residual in (**I**): tissue , nuclei , matrix ; Tubule compartments: nuclei , cytoplasm  and lumen ; capillaries .

Our cohort of renal allograft biopsy samples represents a broad range of histopathology, from normal to various degrees of chronic and active injury. The training (n = 680) and internal test (n = 145) subsets did not differ by demographics or Banff score distributions. The external test set (n = 94), procured from the Department of Pathology and Laboratory Medicine, University of California, Davis School of Medicine, Sacramento, CA, USA, despite different demographics, exhibited a histopathological range akin to the Vilnius cohorts (Supplementary Table S1) except for interstitial inflammation, which was more pronounced in the external set. Summary statistics of the morphometry features extracted by the computational workflow (see Fig. 1 and the Methods section) are presented in Table 1

**Table 1:** Summary statistics for key histological variables used in the analysis of renal cortical tissue.

	N	Mean		sd		Median		Min		Max	
		Original	z-scored	Original	z-scored	Original	z-scored	Original	z-scored	Original	z-scored
tLumen%	680	14.64	0.03	8.85	0.98	12.06	− 0.26	1.38	− 1.44	51.41	4.1
	145	14.75	0.02	9.15	0.95	12.78	− 0.19	1.73	− 1.34	56.43	4.34
	94	10.29	0.17	3.7	0.94	10.46	0.21	2.61	− 1.78	21.19	2.94
tCell%	680	37.38	0.05	10.71	0.96	38.2	0.12	3.98	− 2.95	65.74	2.59
	145	37.4	0.05	10.07	0.99	38.5	0.16	10.93	− 2.56	60.89	2.37
	94	52.53	0.18	10.3	0.87	54.25	0.33	23.82	− 2.25	74.17	2.02
iMatrix%	680	8.32	− 0.07	4.23	0.95	7.42	− 0.28	1.47	− 1.62	36.28	6.23
	145	9.13	0	5.11	1.03	7.45	− 0.33	2.43	− 1.34	26.79	3.55
	94	16.78	− 0.09	9.87	0.99	13.21	− 0.44	5.06	− 1.26	48.01	3.05
iColl%	680	34.05	− 0.04	11.31	0.97	32.34	− 0.19	12.74	− 1.87	80.98	3.98
	145	31.73	− 0.05	11.24	0.97	30.22	− 0.18	13.46	− 1.62	70.45	3.29
	94	14.43	− 0.23	5.26	0.81	12.94	− 0.46	5.08	− 1.67	29.88	2.14
ptc%	680	5.61	0.01	1.84	0.98	5.56	− 0.02	1	− 2.46	10.92	2.85
	145	6.98	− 0.05	1.74	0.98	7.16	0.05	1.63	− 3.06	12.22	2.9
	94	5.96	− 0.15	1.89	0.57	6.22	− 0.07	0.17	− 1.9	9.87	1.03
tSize	680	3283.39	0.08	666.04	0.97	3313.49	0.12	1023.41	− 3.21	5203.45	2.87
	145	3202.76	0.05	691.39	1.03	3240.2	0.11	1249.58	− 2.85	4917.67	2.6
	94	2365.2	0.13	455.68	0.95	2322.91	0.04	1099.8	− 2.51	3593.05	2.7
tCellDen	680	3721.35	− 0.06	834.69	0.9	3591.97	− 0.2	0	− 4.08	7482.03	4
	145	3949.53	− 0.1	804.5	0.93	3854.95	− 0.21	2193.35	− 2.14	7754.82	4.31
	94	4671.25	− 0.21	785.86	0.72	4536.83	− 0.33	3334.85	− 1.43	7280.36	2.19
CTA	680	8.81	*	3.73	*	8.01	*	4.02	*	37.87	*
	145	11.43	*	5.23	*	9.89	*	4.31	*	30.59	*
	94	6.03	*	2.93	*	5.28	*	3.04	*	21.18	*
GlomN	680	17.56	*	9.52	*	15	*	5	*	66	*
	145	23.14	*	14.59	*	19	*	5	*	101	*
	94	16.5	*	9.72	*	14	*	5	*	57	*

Measures include mean, standard deviation, median, minimum, maximum, presented for training (n = 680), internal (n = 145) and external (n = 94) test subgroups.

### PCA and cluster analysis

**Figure 2:**
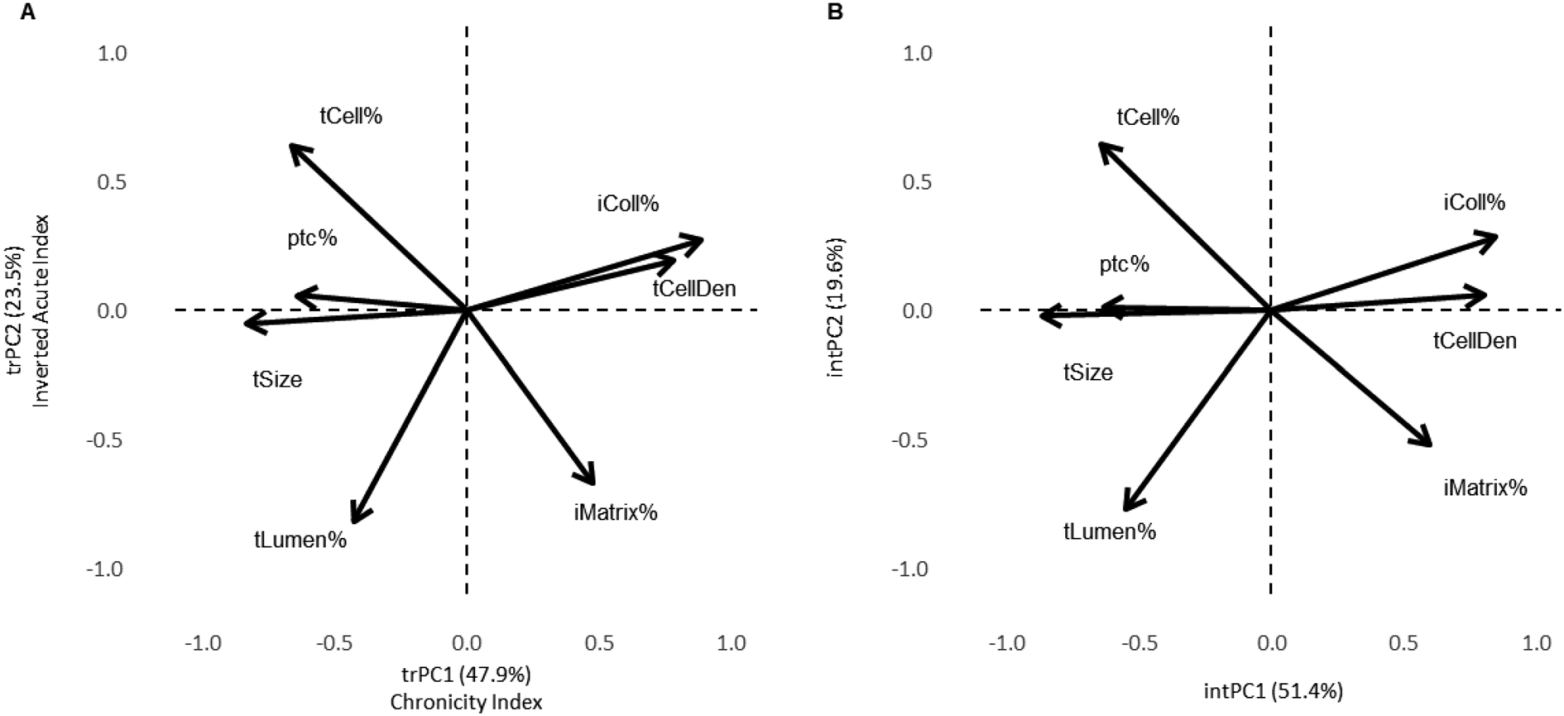
Principal Component Analysis (PCA) plot displaying the projection of 7 features in the training (**A**, n = 680) and internal test (**B**, n = 145) sets onto the first two principal components. Variables are represented by arrows, with the direction and length of each arrow indicating how each variable contributes to the principal components. In (**A**) the axes trPC1 and trPC2 represent training set, while in (**B**) intPC1 and intPC2 represent internal test PCs, revealing similar variance within both datasets.

We applied PCA to explore multivariate associations between the morphometry features in the datasets presented in Table 1 Two principal components were extracted in the training dataset (Fig. 2A). The first component (labelled trPC1 to remind it is the PC1 from the training set) is best interpreted as an integrated Chronicity Index: it is primarily represented by strong positive loadings of fibrillary collagen area fraction (iColl%) and density of tubular cell nuclei (tCellDen) and by negative loadings of tubular size (tSize) and tubular cell area (tCell%); this pattern represents expansion of the fibrillary component in the interstitium along with shrinkage of the tubular component. Noteworthy, the latter is accompanied by a decrease in peritubular capillary fraction (ptc%). The second component (similarly labelled trPC2) is represented by negative loadings of the tubular lumen fraction (tLumen%) and non-fibrillary matrix fraction (iMatrix%) with a positive loading of the tubular cell area fraction (tCell%); therefore, it can be interpreted as an feature of an “inverted” acute tubulointerstitial injury (Inverted Acute Index) represented by flattening of tubular epithelial cells along with widening of tubular lumen and non-fibrillary matrix area (interstitial edema). By definition, the Chronicity Index and Inverted Acute Index are linearly independent and can be regarded as integrated quantitative indices of chronic and acute tubulointerstitial transformations, respectively. A similar PCA pattern was reproduced in the internal test set (Fig. 2B) which indicates robustness of the extracted features and supports the interpretation of PCA components.

**Figure 3:**
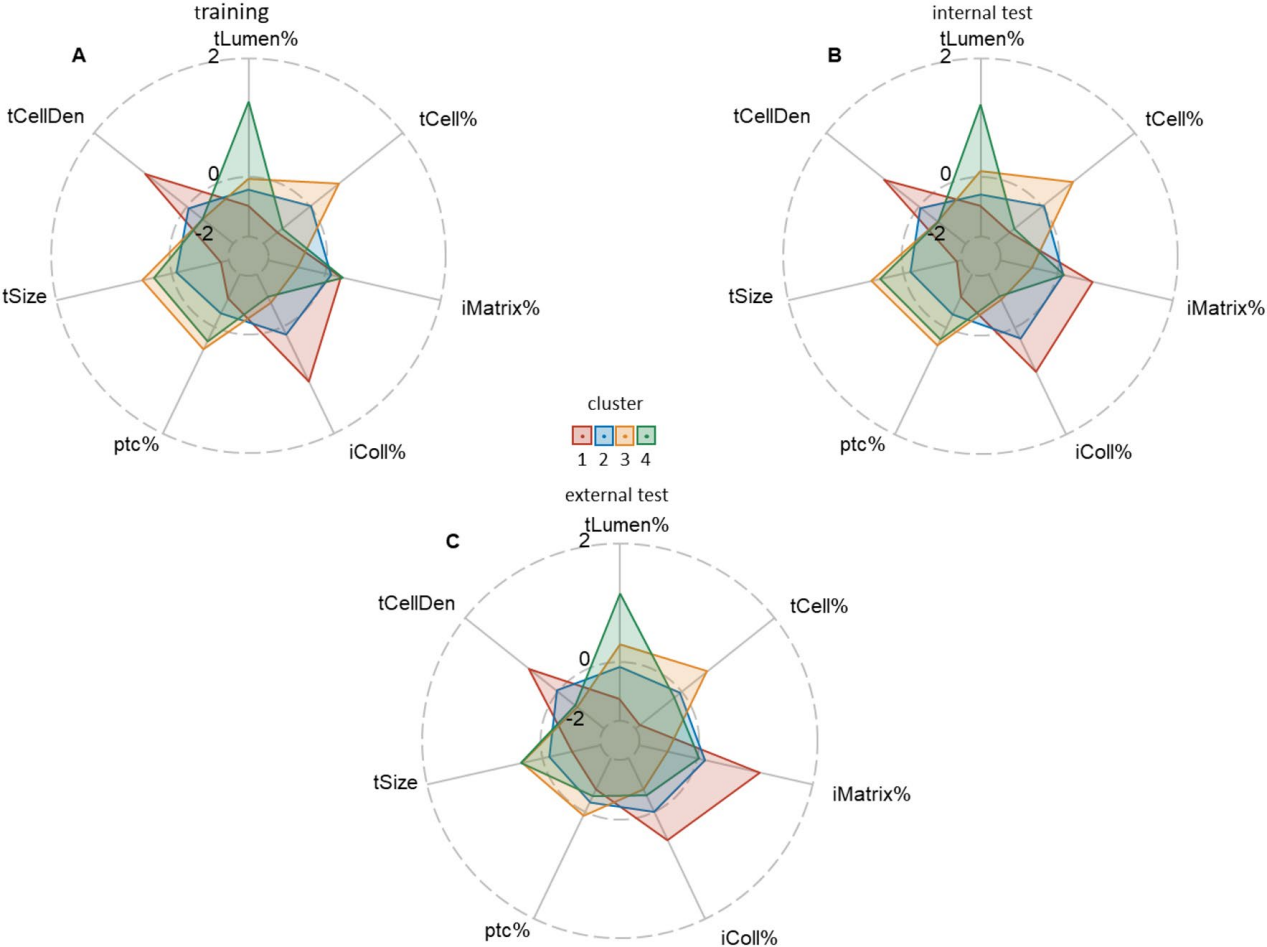
Faceted radar plot illustrating cluster analysis based on extracted features. Each facet represents a distinct cluster in training (**A**, n = 680), internal test (**B**, n = 145) and external (**C**, n = 94) z-scored sets, with multiple axes displaying the variables considered in the analysis. The axes are not rescaled to maintain the original scale of the data: cluster 1 contains biopsy cases with the highest fibrillary collagen area fraction and tubular cell density; cluster 2 – average values of all the indicators; cluster 3 the highest area fractions of tubular size, peritubular capillaries, and tubular cells; cluster 4 – the highest tubule lumen area along with low tubular cell area fraction accompanied by expansion of non-fibrillary matrix area.

To classify potential phenotypes of acute and chronic transformations, we performed an unsupervised K-means clustering analysis of the biopsy cases in the space of the seven selected tubulointerstitial indicators. The profiles of the four extracted clusters reveal peculiar patterns in the training and both internal and external test sets (Fig. 3). Based on these profiles, cluster 3 represents relatively intact tubulointerstitium, while clusters 2 and 1 contain biopsy cases with increasing transformation towards chronicity (IFTA). Cluster 4 represents features of acute tubular damage and interstitial expansion by the non-fibrillary matrix in the context of low chronicity indicators.

**Figure 4:**
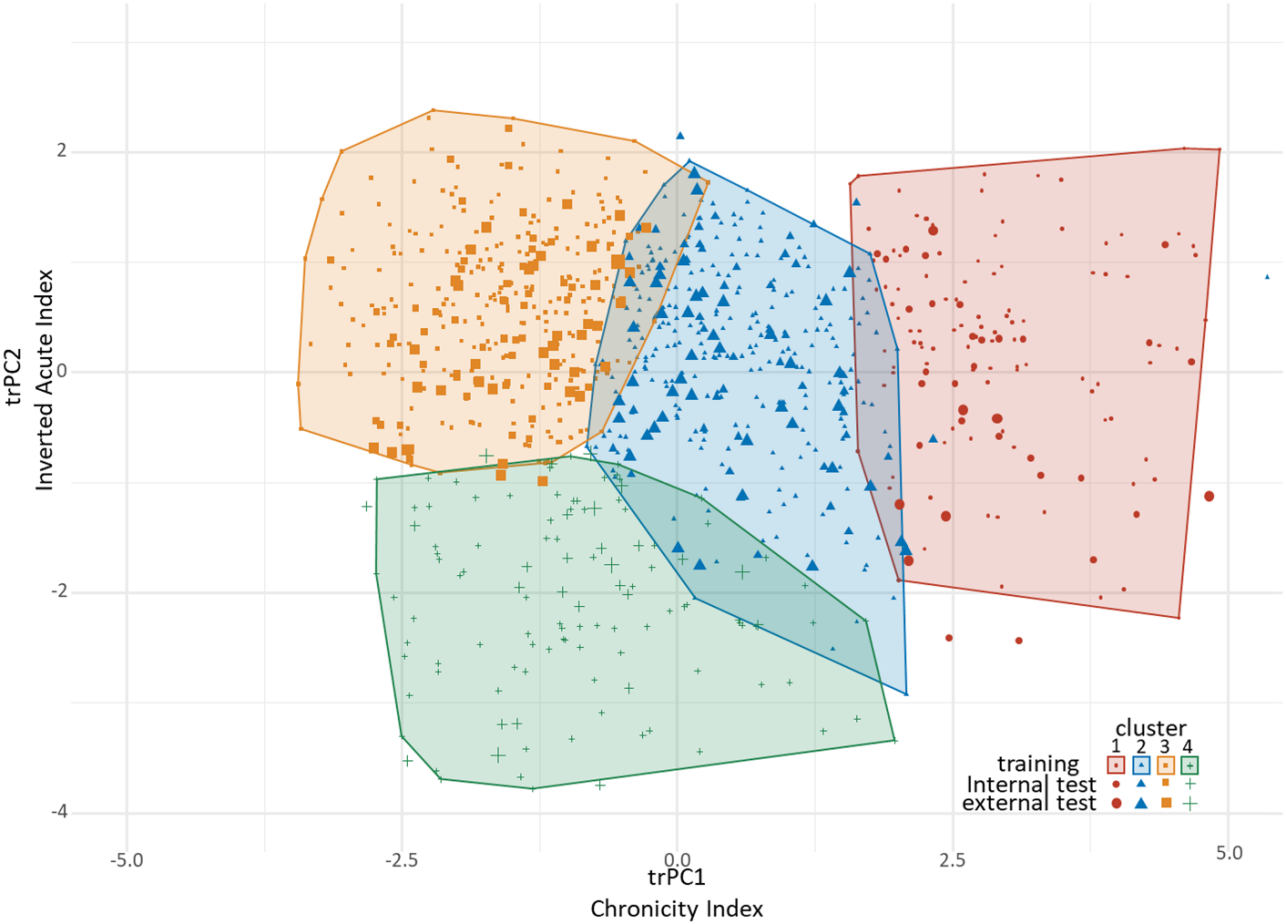
Clusters of biopsy cases plotted against the Training first two principal components derived from the PCA analysis of the extracted features; cluster boundaries are generated for training set(n = 680), cases from the internal test set (n = 145) are represented by medium size symbols, cases from the external test set (n = 94) are represented by largest symbols. Both test sets are projected onto the training PCA space.

For visualization purposes, the Chronicity Index and Inverted Acute Index (their values for both testing sets computed based on the trPC1 and trPC2 data) were plotted to reveal the distribution of the biopsy cases in the four clusters (Fig. 4). As above, the largest cluster 3 represents a relatively intact tubulointerstitial architecture (low Chronicity Index and high Inverted Acute Index), clusters 2 and 1 contain cases with intermediate and high Chronicity Index values (IFTA transformation), respectively. Meanwhile, cluster 4 represents a relatively small number of cases with low Inverted Acute Index and low-to-intermediate Chronicity Index. This distribution of clusters reveals that acute and chronic components of tubulointerstitial injury can be distinguished, while in the context of advanced chronic lesions (high Chronicity Index), the acute component is less pronounced or retrievable.

**Figure 5:**
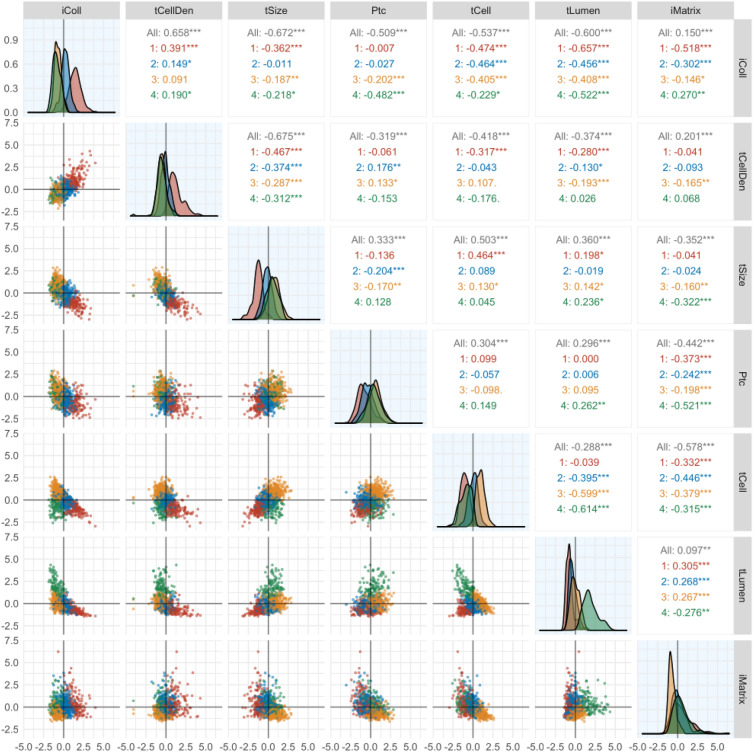
Distribution and pairwise correlation analyses of the features in whole dataset and within the individual clusters (1–, 2– , 3– , 4– ) in training and test data sets combined (n = 825). Scatter plots (lower panel) showing relationship between features in different clusters. Density plots (color ) of features across different clusters. Pearson correlation coefficients (upper panel) for the relationship between features in whole dataset and across different clusters.

To highlight differences between the feature variables within the clusters that may be obscured in the whole dataset, pairwise correlations and distributions of the 7 indicators within the 4 clusters were plotted in Fig. 5. The three IFTA progression clusters (clusters 3-2-1) display linear associations of the variables consistent with the Chronicity Index pattern (columns 1–5) in the whole data. The Inverted Acute Index attributes—tubular lumen (tLumen%) and tubular cell mass (tCell%)—reveal weak negative correlation in the whole dataset and no correlation in cluster 1 (high IFTA); however, they reveal negative correlations in clusters 3 (relatively intact) and 4 (acute injury), r = − 0.599 and r = − 0.614, respectively. Similarly, the fractions of non-fibrillary matrix and fibrillary collagen (iMatrix% and iColl%, respectively) do not correlate (r = 0.150) in the whole data set, while they reveal negative correlations only in clusters 1 (advanced IFTA) and 2 (moderate IFTA), r = − 0.518 and r = − 0.302, respectively, and becomes positive in “acute tubular injury” cluster 4. The distributions of the indicators within the clusters also illustrate the discriminatory value of the individual morphometric indicators; for example, the density plots of high tubular lumen fraction (tLumen%) illustrate that it is a distinct feature of cluster 4 (acute), with little overlap within cluster 3 (relatively intact), and even less within clusters 1 and 2 (chronic).

**Figure 6:**
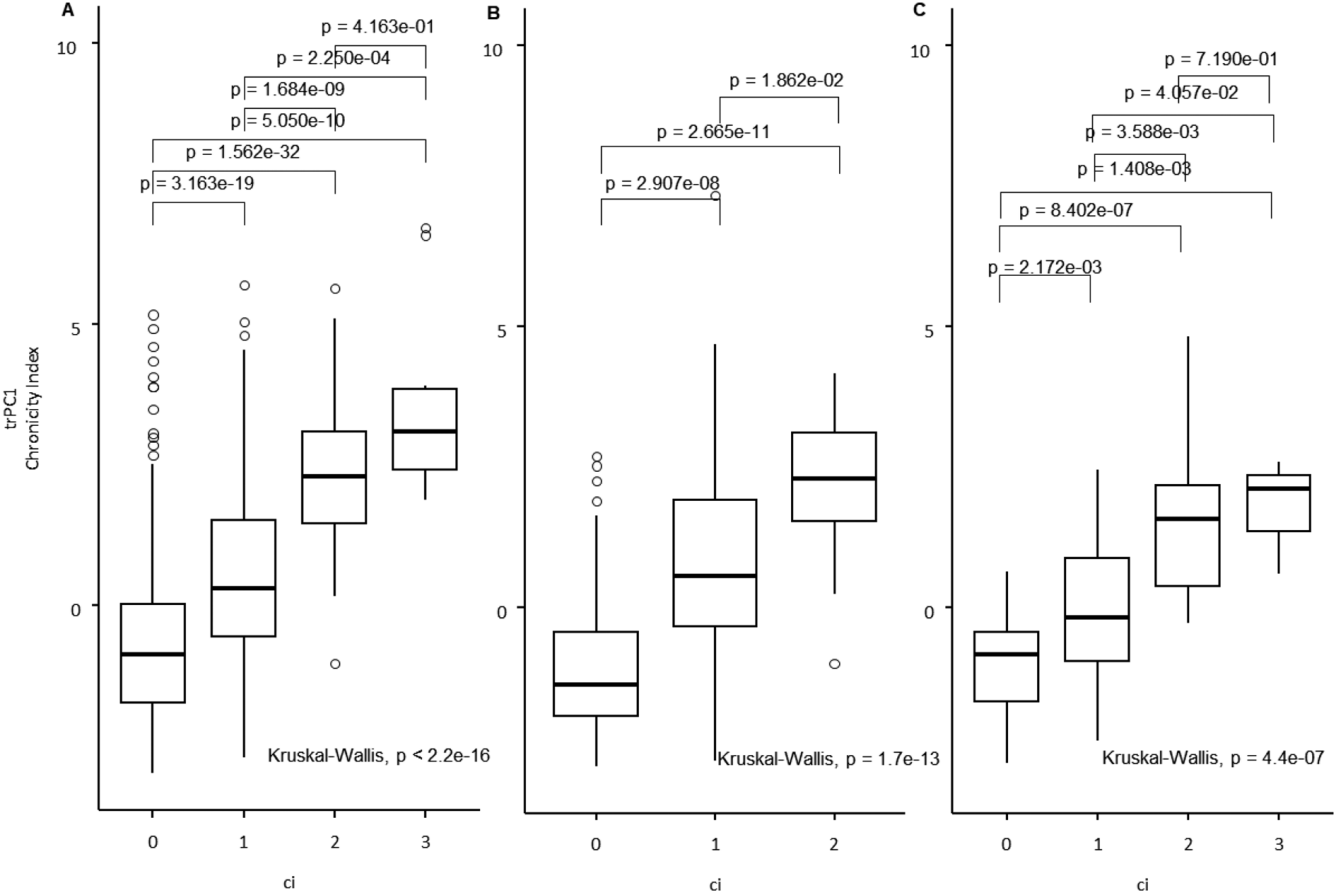
Boxplot showing the distribution of Chronicity Index trPC1) for ci (interstitial fibrosis) (**A**, **B**, **C**) in training (**A**) and both internal and external test (**B** and **C**) sets. Statistical significance was assessed using Dunn's test for pairwise comparisons and Kruskal–Wallis test for overall group differences.

To test our hypothesis that trPC1 represents an integrated Chronicity Index, we tested its associations with a semiquantitative assessment by Banff classification scores from pathology reports. In training and both internal and external test sets, the Chronicity Index was positively associated with Banff ci score (a semi-quantitative assessment of interstitial fibrosis), Fig. 6. We did not find any significant associations between the Inverted Acute Index and Banff scores.

**Figure 7:**
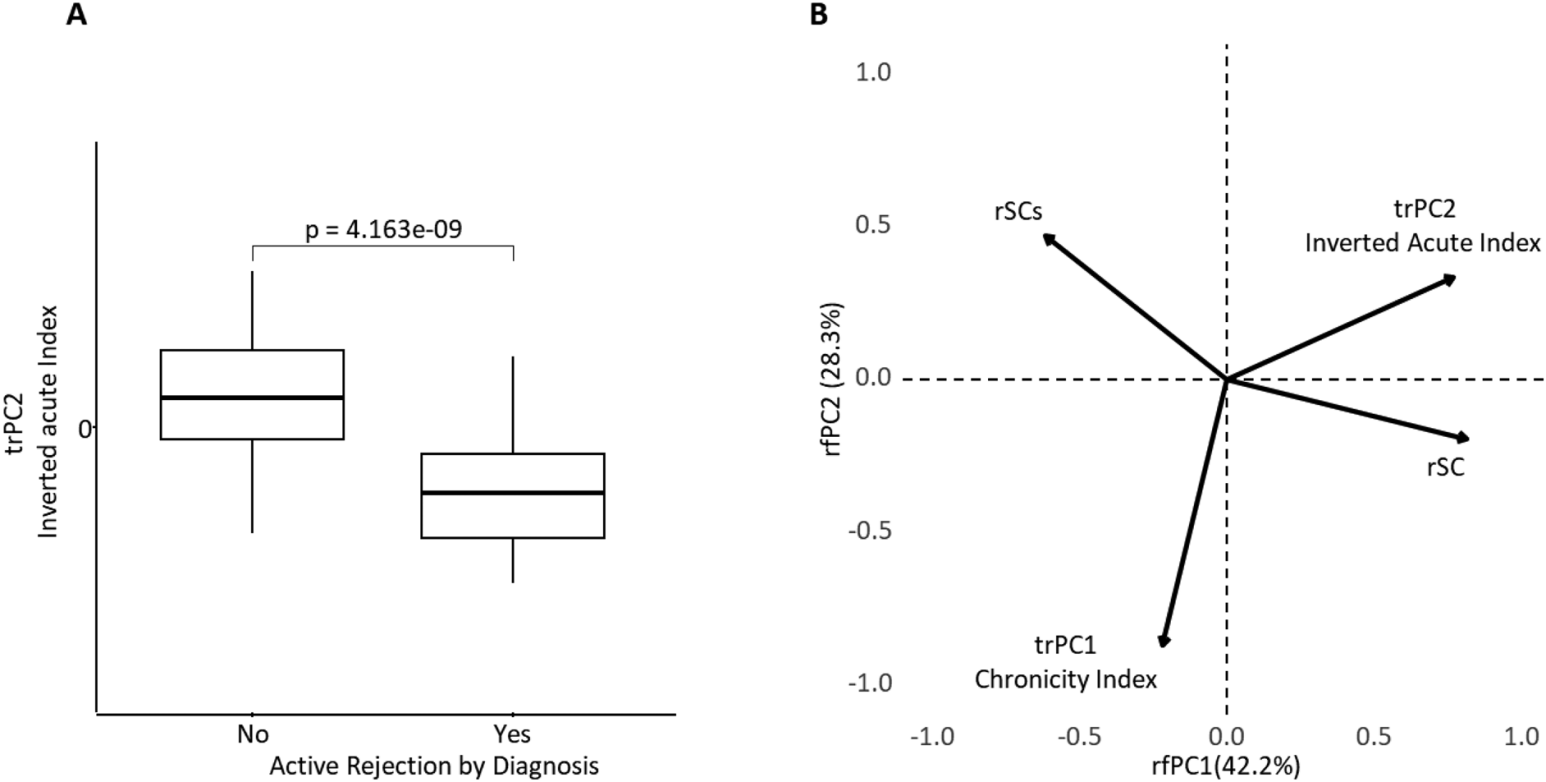
(**A**): Boxplot illustrating the distribution of trPC2—Inverted Acute Index—stratified by Active Rejection status by pathology diagnosis. Dunn's test was used for pairwise statistical comparisons. (**B**): Principal Component Analysis displaying interaction of the Inverted Acute and Chronicity Indices, computed for the external set along with reciprocal serum creatinine indicators (rSCs and rSC), n = 44. The extracted principal components include the renal function loadings, therefore, are labeled as rfPC1 and rfPC2.

We further investigated the clinical relevance of the model outputs in the external set. Significant association between the Inverted Acute Index and diagnostic Banff categories of acute rejection was found: the lower tertile of the Inverted Acute Score range contained the highest frequency (65.7%) of acute rejection diagnostic Banff categories compared to the middle and higher tertiles, 28.6% and 5.7%, respectively (*p* < 0.0001, n = 94). Similarly, the Inverted Acute Index was lower (*p* < 0.0001) in biopsies with pathology diagnosis of cell- and/or antibody-mediated acute rejection (Fig. 7A).

In the external set, we investigated associations of the Chronicity Index and Inverted Acute Index with renal function at the time of biopsy represented by reciprocal Serum Creatinine (rSC) and rate of progression of renal insufficiency during the follow-up, represented by reciprocal Serum Creatinine slope (rSCs) computed by regression. To account for possible multiple interactions between the variables, we performed PCA (Fig. 7B) that extracted PC1 (labeled as rfPC1 – renal function PC1) characterized by strong positive loadings of Inverted Acute Score and rSC at the time of the biopsy (both reflecting preserved renal morphology and function) along with moderate negative loadings of rSCs (relatively less rapid loss of renal function after the biopsy with acute injury); the latter suggests potential (more probable) recovery of renal function after an acute injury episode diagnosed in the biopsy which may positively affect the rSCs indicator. The rfPC2 is represented by strong negative loadings of Chronicity Index and moderate positive loadings of rSCs suggestive of more rapid (more probable) deterioration of renal function after the biopsy with higher Chronicity Index (as interpreted by inverted values of trPC2 score). Importantly, Inverted Acute and Chronicity indices are only weakly associated in this PCA model, thus supporting their independence as integrated morphometry-based indicators. Pairwise correlations of rSC and rSCs values and primary morphometry variables are presented in the Supplementary Table S2.

## Discussion

In this study, we present a DIA data-driven computational method to extract and quantify acute and chronic transformations within the tubulointerstitial compartment of the renal cortex. The continuous data-based morphometric indicators enable multivariate analyses to assess the acute and chronic components of the tubulointerstitial injury. The method provides continuous principal component scores for the components as well as informative value of the seven individual morphometry indicators. We also demonstrate that the biopsy cases can be classified into relatively homogenous clusters (phenotypes); furthermore, the clusters reveal different intrinsic associations and distributions of the morphometry indicators. The model was reproducible in two independent biopsy data sets and provided relevant associations with the Banff scores from pathology reports as well as serum creatinine profiles of the patients.

Our method is based on a computational pipeline which starts with an automated segmentation of the main renal tissue compartments by DL algorithms. Previous studies have reported successful segmentation of glomeruli, tubules, peritubular capillaries, and other components^21,22,32^. Similar to Madabhushi et al.^22^, we developed a set of independent one-versus-all classifiers to segment and classify the main kidney structures including tubules, peritubular capillaries, glomeruli, and arteries. Our rationale for applying independent classifiers was based on (and supported by our initial experiments) the fact that the typical size and detail of the components to segment is highly variable and can be best achieved by training the DL models at relevant resolutions. For example, Madabhushi et al.^22^ achieved optimal segmentation of peritubular capillaries on WSI at 40 × magnification. Our study was performed on sections stained by modified Picrosirius red and scanned at 20 × objective magnification. We trained DL at high resolution settings to get optimal results for peritubular capillaries; meanwhile, large structures – glomeruli, renal cortex—were better segmented by classifiers trained at lower resolutions. Such approach allowed focusing on different features of the kidney microarchitecture and was independently optimized according to the needs of subsequent analyses. In addition, we took an advantage of utilizing the outputs from multiple classifiers for quality assurance of the final segmentation results: overlapping outputs from any classifiers indicated potential errors and were used for error correction, for example, peritubular capillaries detected within the glomerular area (most common error) were removed from further analyses.

We used the DL-based segmentation to sample the renal structures for extraction of explicit measurable features by additional DIA for further statistical modeling, similar to the recent study by Holscher et al.^31^. Based on an initial exploratory investigation, we decided to focus on 7 indicators that together represent major morphometric transformations in acute and chronic pathology of the tubulointerstitial compartment. Five indicators represent area fractions of tubular lumen (ultrafiltrate), tubular cell mass, fibrillary and non-fibrillary matrix of the interstitium, and peritubular capillaries. Supplemented by the mean tubular area and tubular cell (nuclei) density, it forms a comprehensive set of indicators that represents global tubulointerstitial phenotype transformations. In contrast to Holscher et al.^31^, instead of glomerular, tubular, and vascular morphometry testing, we focused on multivariate modeling of the tubulointerstitial morphometrics as a major known pathology correlate of acute and chronic renal impairment. Although we performed our study on transplanted kidney biopsy WSI, the same principles may apply to native kidney pathology.

We succeeded in an automated segmentation of renal cortex and medulla tissue with a good accuracy, indirectly illustrated by the fact that a small proportion of all glomeruli were segmented in the “medulla”: 1.4% and 1.1% in the training and test sets, respectively (data not shown). To the best of our knowledge, our DL algorithm is the first one trained to segment the renal cortex tissue independently of the presence of glomeruli. In contrast, Farris et al.^9^ established a pipeline for renal cortex segmentation based on automated identification of glomeruli with subsequent selection of surrounding regions of interest (ROIs).

Our study presents a proof of concept that renal biopsy DIA-based computational models, using quantitative data for multivariate statistics allows extraction and quantification of acute and chronic components of the tubulointerstitial injury. Recently, Vaulet et al.^30^ also proposed a data-driven method to identify chronic allograft phenotypes, but they instead performed a clustering of semiquantitative Banff scores and supplementary data. They did demonstrate a potential of their multivariate model for predicting kidney allograft outcomes, but it is still sensitive to inter- and intra-observer variability of histologic scoring. This concern is alleviated using DIA-based data, and one might expect that the quantitative nature of the extracted data may lead to more informative computational models. For instance, our findings demonstrate the importance of multivariate modeling: intrinsic associations between the morphometry variables were different within the clusters which implies qualitative differences between the tubulointerstitial phenotypes identified; importantly, these associations were obscured in the common dataset.

Our study contains limitations. Although we were able to reproduce our computational model in two independent (internal and external) data sets, it requires further validation studies. Validation against Banff scores is of limited value, partially due to the semiquantitative nature of the pathology scoring. The performance of the model for predicting clinical data and outcomes is needed in appropriate and large-scale data sets to ensure their generalization across different laboratories and patient populations. Although we found relevant associations of the model outputs with acute rejection diagnoses and serum creatinine profiles, more comprehensive datasets would be needed for further testing. Finally, the model provides global assessment of the tubulointerstitium of renal cortex while assessment of intratissue heterogeneity of the transformations could provide a more granular assessment; this will be explored in further studies. Similarly, including immune cell distributions would lead to a comprehensive assessment of renal allograft biopsy.

In conclusion, we propose a computational model to quantify acute and chronic transformations of the tubulointerstitial compartment of the renal cortex. The computational pipeline is based on DL-based segmentation of the relevant kidney structures with subsequent DIA-based feature extraction and quantification. The principles of the model construction can be applied for development of integrated computational applications for native and transplanted kidney pathology.

## Materials and methods

WSI of slides (n = 1021) stained by modified Picrosirius red during routine kidney allograft biopsy diagnosis were used for the training (n = 852) and internal test subsets (n = 172) in this study. One indication (n = 786) or protocol (n = 238) biopsy containing at least 5 glomeruli per unique patient was randomly selected from a database containing a total of 1021 biopsied patients along with their pathology report data (age, gender, Banff score). The biopsy diagnosis was performed at the National Centre of Pathology, Affiliate of Vilnius University Hospital Santaros Klinikos. The study was performed in accordance with relevant guidelines and regulations and was approved by the Vilnius Regional Biomedical Research Ethics Committee of Vilnius University (reference number: 2019/6–1148-637, date 2019–06-18). Patient informed consent was waived by the Vilnius Bioethics Committee according to the International Ethical Guidelines for Health-related Research Involving Humans^33^. An external test set (n = 150), comprising both indication (n = 75 and protocol (n = 75) biopsies along with patient data (Banff categories and scores from pathology reports, serum creatinine levels at the biopsy and follow-up period) was used as approved by the Institutional Review Board of the University of California, Davis.

Routine formalin-fixed paraffin-embedded tissue techniques were used in the diagnostic routine. Briefly, sections of 2-μm thickness were stained with modified Picrosirius red staining, which combines Picric acid-Sirius Red solution for staining the cell cytoplasm and collagen fibers and Mayer’s Hematoxylin for staining the cell nuclei and basophilic tissue elements, see (Fig. 1A). The staining procedure involves the steps of deparaffinization of the sections, hydration in distilled H_2_O, 0.1% Picric acid solution (1 h at room temperature), 0.5% Acetic acid solution (rinse 2–3 times), Mayer's hematoxylin (5 min), running tap H_2_O (3 min), dehydration with isopropyl alcohol and clarification with xylene. The final results are evaluated by color indicators: red for collagen, light yellow for cell cytoplasm, and blue for cell nuclei. IHC for CD34 was performed using EnVision FLEX high-sensitivity visualization system on Dako Autostainer Link staining system (DAKO, Glostrup, DK). The CD34 monoclonal mouse antibody (clone QBEnd10; DAKO, Glostrup, DK) was applied at a 1:25 dilution for 30 min, followed by EnVision FLEX + Mouse (Linker) for 15 min. Finally, the sections were developed in DAB at 37 °C for 10 min, counterstained with Mayer’s hematoxylin and mounted. The slides were scanned at the time of diagnosis with Aperio AT2 DX with × 20 objective and pixel size of 0.5 μm (Leica Biosystems, Wetzlar, Germany).

### Image analysis

DIA of the WSI was performed using HALO version 3.5.3577 and HaloAI version 3.5.3577 (Indica Labs, Corrales, NM). The Densely Connected Convolutional Networks architecture implemented in HaloAI support sampling network input patches at a specific pixel resolution ( μm) and filtering the segmented objects/regions by a minimum area ( μm^2^). By using individual classifiers these parameters can be optimized when training classifiers for different kidney histology compartments.

A classifier for renal cortex versus medulla (Fig. 1B and C) and a set of independent one-versus-all classifiers were developed to segment and classify arteries, arterioles and veins (Fig. 1D), peritubular capillaries (PTC) (Fig. 1E), glomeruli (Fig. 1F), tubules (Fig. 1D). By pilot experiments, the following pixel resolution and minimum areas were chosen: healthy and sclerosed glomeruli (1  μm/px, 500  μm^2^), PTC (0.25  μm/px,40  μm^2^), tubules (0.25  μm/px, 200  μm^2^), arteries and veins (2  μm/px, 1000  μm^2^), cortex and medulla (4  μm/px, 1000  μm^2^). For the PTC classifier, images of slides re-stained by immunohistochemistry for CD34 (endothelial biomarker) were used to produce an initial set of annotations. To train all the classifiers the “human-in-the-loop machine learning approach”^34^ was used to generate high numbers of annotations. The annotations were produced or corrected by an image analysis researcher (RA) under guidance of an experienced nephropathologist (AL). Overall, 16,296 annotations were generated in 200 WSI. Segmentation validation definitions and metrics are presented in Supplementary Table S3 and example of PTC validation in Supplementary Figure .

The segmentation output masks produced by the individual classifiers for glomeruli, tubules, peritubular capillaries, arteries, arterioles and veins, were subsequently compiled into a single mask (Fig. S2^1^H). This enabled review, quantification and intersections regions of class overlaps for quality assurance of the segmentation and classification. Examples of compiled classifiers and overlaps are presented in Supplementary Figure S1. For further analyses, overlaps were resolved by preset prioritization rules to correct for typical errors. Most commonly, PTC were “found” in the area of glomeruli and were removed from further analyses by giving a higher priority to glomeruli and other tissue classes. After computational removal of the areas of glomeruli, PTC, tubules, arteries, and veins, the remaining cortical tissue area was presumed to represent the interstitium. This “residual” tissue was further subjected to feature extraction by a classifier to segment red fiber collagen, white–pink non-fiber matrix, and blue nuclei evaluated (Fig. 1I). Tubular compartments (nuclei, cytoplasm, lumen) were further assessed using area quantification (AQ) based on relevant color assessment, blue, light grey and white, respectively, (Fig. 1J).

### Sampling criteria

In addition to the initial tissue sampling criterion (> 5 glomeruli by pathology report), we applied a criterion of at least 4 square millimeters of cortical tubulointerstitial area (CTA), after excluding the glomeruli and arteries. This ensured that the tubulointerstitial compartment was well-represented for further analyses independent of potentially crowded glomeruli in some biopsies. In the external set, a criterion of at least 3 square millimeters of CTA was applied due to relatively small amount of tissue in the sections. After implementing this criterion along with the requirement for a minimum of 5 glomeruli, the dataset was reduced to 94 cases from the initial 150.

### Feature extraction

For a comprehensive morphometric representation of the tubulointerstitial compartments of renal cortex, we were able to compute 56 compartment-specific features. To avoid an impact of highly variable tissue sample size, relative area indicators of the renal cortex compartments were selected that represent a Cortical Tubulointerstitial Area (CTA) defined as a global sum of the areas of tubular lumen (tLumen%), tubular cells (tCell%), peritubular capillaries (ptc%), and interstitium represented by fibrillary collagen (iColl%) and non-fibrillary matrix (iMatrix%). In addition, 2 global indicators were selected to characterize tubular morphology: density of cell nuclei in tubules (tCellden) and mean area of tubules (tSize). The variable definitions are presented in Table 2.

**Table 2:** Detailed list of variables, along with their mathematical formulas and definitions, used in the quantitative analysis of renal cortical tissue.

Variable	Formula	Definition
CTA	$$\begin{gathered} = biopsy \;area - glomeruli \;area - arteries \;area \hfill \\ = tubular \;lumen \;area + tubular \;cell \;area \hfill \\ + peritubular \;capillaries \;area \hfill \\ + fibrillary \;collogen \;area \hfill \\ + non\hbox{-}fibrillary \;matrix \;area \hfill \\ \end{gathered}$$	Cortical tubulointerstitial area
tLumen%	$= \frac{tubular \;lumen \;area}{{{\text{CTA}}}} \times 100$	Percentage of tubular lumen area
tCell%	$= \frac{tubular \;cell \;area}{{{\text{CTA}}}} \times 100$	Percentage of tubular cells area
ptc%	$= \frac{peritubular \;capillaries \;area}{{{\text{CTA}}}} \times 100$	Percentage of peritubular capillaries area
iColl%	$= \frac{ fibrillary \;collagen \;area}{{{\text{CTA}}}} \times 100$	Percentage of fibrillary collagen area
iMatrix%	$= \frac{{non\;\hbox{-}fibrillary \;matrix \;area}}{{{\text{CTA}}}} \times 100$	Percentage of non-fibrillary matrix area
tSize	$= \frac{tubular \;lumen \;area + tubular \;cells \;area}{{{\text{CTA}}}} \times 100$	Mean area of tubules
tCellden	$= \frac{{N\left( {cells} \right)}}{tubular \;area} \times 100$	Densities of cells in tubules

The table includes metrics related to tubular structure, peritubular capillaries, and matrix components, all calculated relative to the Cortical Tubulointerstitial Area (CTA).

Statistical analyses were performed using RStudio (2022.12.0). To enable comparison of features with different scales, the data were converted into z-scores. Variables were checked for normality of distribution by Shapiro–Wilk statistic; since the data was non-normally distributed, Dunn's test for pairwise multiple comparisons and Kruskal–Wallis test for more than two groups comparisons were used. To explore feature relationships and select the most relevant ones, PCA was used. The transformation defined by the training PCA model was applied to the testing datasets. In other words, testing set PCA scores represent the data in the reduced dimensional space defined by the principal components derived during the PCA fitting process on the training dataset. K-means clustering was employed to explore potential classifications of the biopsy cases, testing a range of cluster numbers to identify the most meaningful division. The prediction function of K-means, obtained in the training set, was applied to the test set to evaluate clustering reproducibility. Pairwise correlation analysis was performed for all features within each individual cluster. Reciprocal Serum Creatinine slope (rSCs) was calculated individually for each patient using linear regression on at least 4 consecutive (Reciprocal Serum Creatinine (rSC) measurements against the time after the biopsy (n = 44).

### Supplementary Information

Supplementary Figure S1.

Supplementary Figure S2.

Supplementary Table S1.

Supplementary Table S2.

Supplementary Table S3.

## Data Availability

The data that support the findings of this study are available from the corresponding author upon reasonable request.
