# Supplementary material for: Computational pathology model to assess acute and chronic transformations of the tubulointerstitial compartment in renal allograft biopsies
Source: Sci Rep. 2024 Mar 4;14:5345. doi: 10.1038/s41598-024-55936-3 (PMC10912734; doi:10.1038/s41598-024-55936-3)
Supplement: Supplementary file 1 — Supplementary Figure S1. [file 41598_2024_55936_MOESM1_ESM.docx]

*
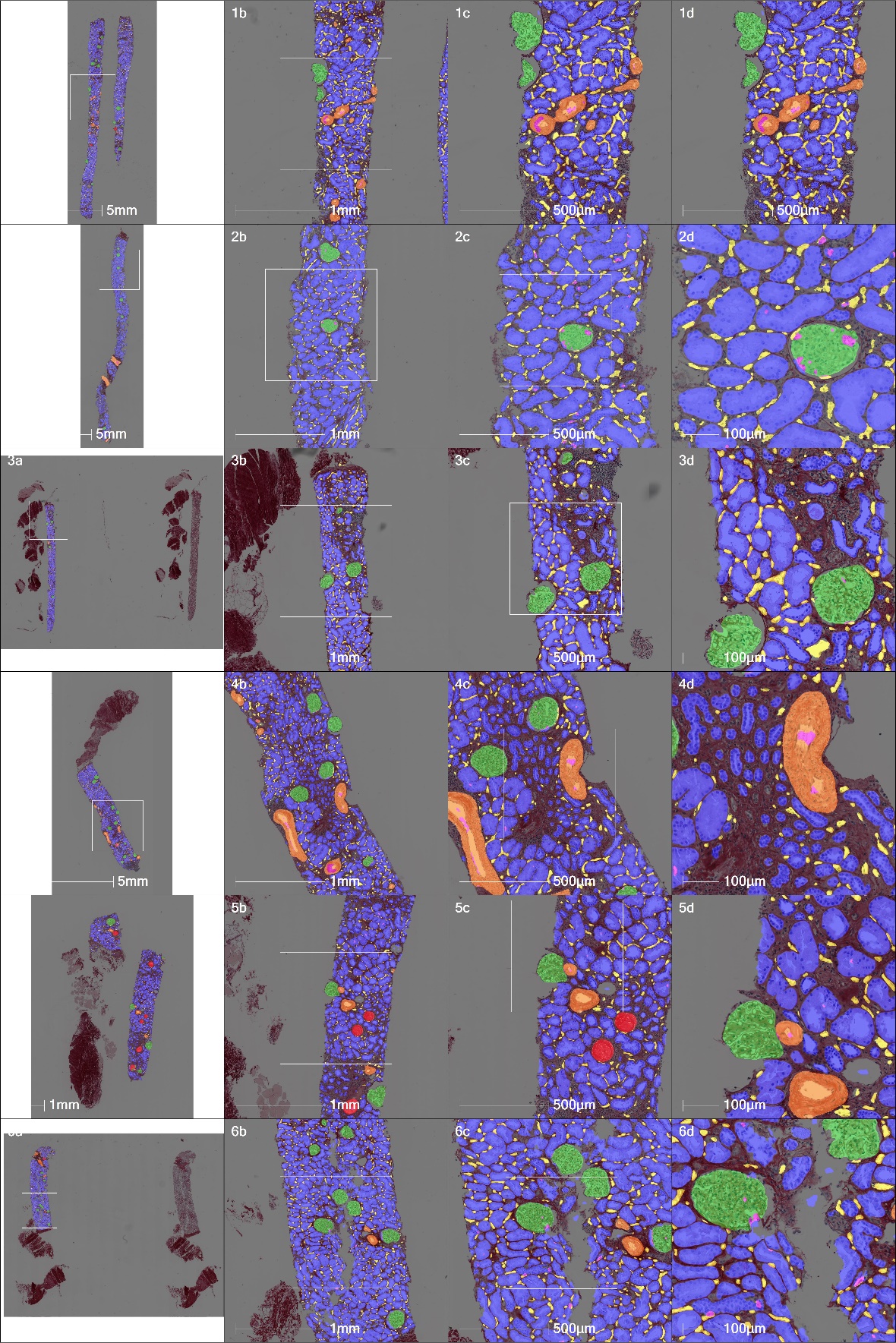
*

Supplementary Figure S1 Segmentation overlay of 6 cases demonstrating the identification and overlap of renal structures by five distinct classifiers at different resolution. Veins and arteries are marked in orange, capillaries in yellow, glomeruli in green, sclerotic glomeruli in red, tubules in blue, and areas of overlap are indicated in pink. This multicolor mapping provides a detailed depiction of the spatial relationships and interactions between various renal compartments.
