# Supplementary material for: Computational pathology model to assess acute and chronic transformations of the tubulointerstitial compartment in renal allograft biopsies
Source: Sci Rep. 2024 Mar 4;14:5345. doi: 10.1038/s41598-024-55936-3 (PMC10912734; doi:10.1038/s41598-024-55936-3)
Supplement: Supplementary file 2 — Supplementary Figure S2. [file 41598_2024_55936_MOESM2_ESM.docx]

*
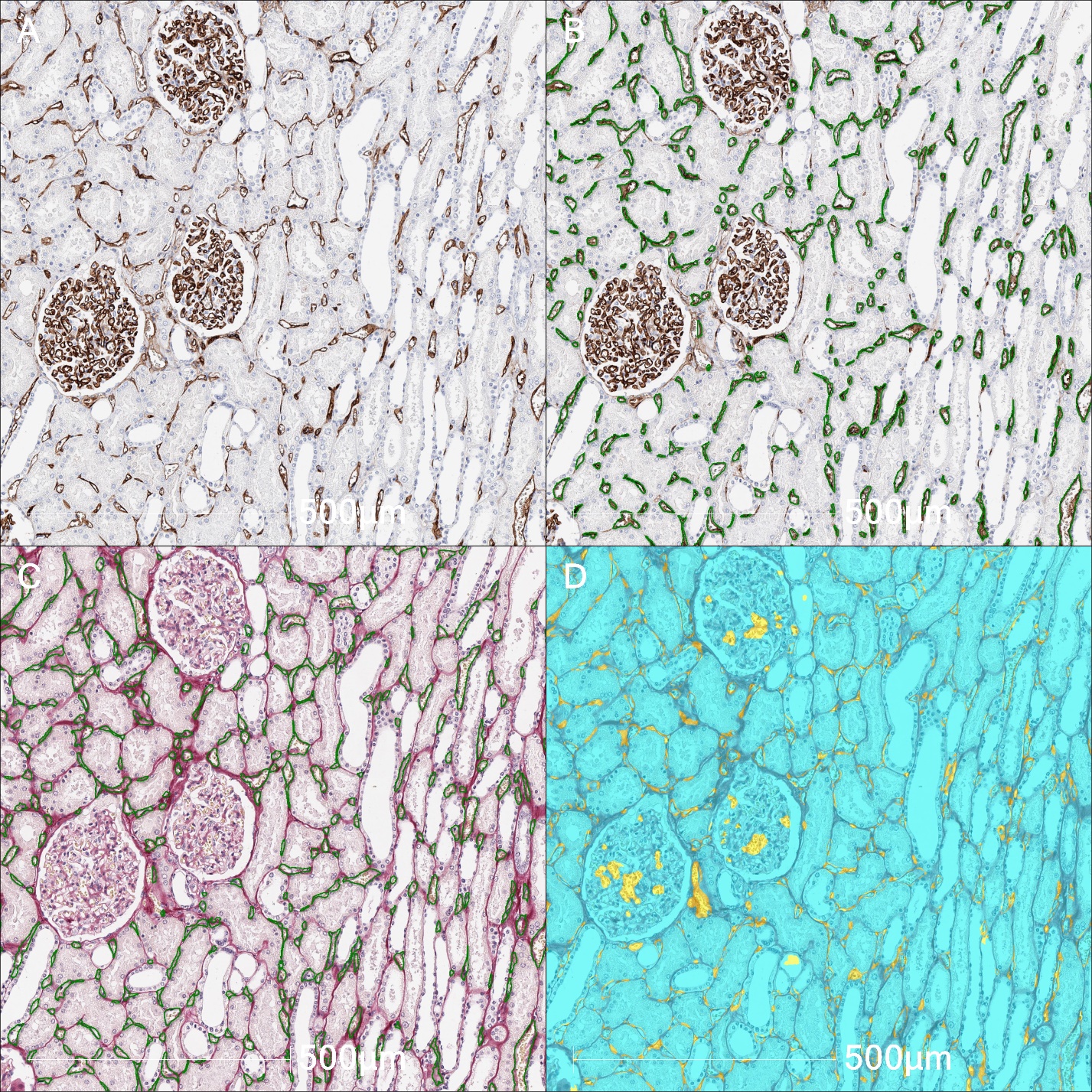
*

Supplementary Figure S2 Validation of capillary segmentation. Panel A displays the original CD34-stained histological image of renal tissue. Panel B shows the same image with added annotations of capillaries based on immunohistochemistry results. Panel C demonstrates the transfer of these capillary annotations to a Picrosirius red-stained image. Panel D presents the segmentation validation results, with cyan indicating correct classifications and yellow highlighting misclassifications.
