# Supplementary material for: Computational pathology model to assess acute and chronic transformations of the tubulointerstitial compartment in renal allograft biopsies
Source: Sci Rep. 2024 Mar 4;14:5345. doi: 10.1038/s41598-024-55936-3 (PMC10912734; doi:10.1038/s41598-024-55936-3)
Supplement: Supplementary file 3 — Supplementary Table S1. [file 41598_2024_55936_MOESM3_ESM.docx]

| Characteristic | Results | | | Characteristic | Results | | |
| --- | --- | --- | --- | --- | --- | --- | --- |
|  | N = 680 | N = 145 | N = 94 |  | N = 680 | N = 145 | N = 94 |
| g (Glomerulitis) | | | | ci (Interstitial fibrosis) | | | |
| 0 | 532 (78%) | 111 (77%) | 68 (72%) | 0 | 393 (58%) | 80 (55%) | 40 (43%) |
| 1 | 118 (17%) | 30 (21%) | 11 (12%) | 1 | 215 (32%) | 45 (31%) | 40 (43%) |
| 2 | 27 (4.0%) | 4 (2.8%) | 9 (9.6%) | 2 | 61 (9.0%) | 20 (14%) | 11 (12%) |
| 3 | 2 (0.3%) |  | 6 (6.4%) | 3 | 11 (1.6%) |  | 3 (3.2%) |
| Unknown | 1 |  |  | ct (Tubular atrophy) | | | |
| cg (Transplant glomerulopathy) | | | | 0 | 346 (51%) | 63 (43%) | 40 (43%) |
| 0 | 552 (82%) | 121 (83%) | 82 (87%) | 1 | 280 (41%) | 62 (43%) | 40 (43%) |
| 1 | 52 (7.7%) | 7 (4.8%) | 5 (5.3%) | 2 | 45 (6.6%) | 18 (12%) | 11 (12%) |
| 2 | 41 (6.1%) | 5 (3.4%) | 6 (6.4%) | 3 | 7 (1.0%) | 2 (1.4%) | 3 (3.2%) |
| 3 | 28 (4.2%) | 12 (8.3%) | 1 (1.1%) | Unknown | 2 |  |  |
| Unknown | 7 |  |  | v (Intimal arteritis) | | | |
| mm (Mesangial matrix expansion) | | | | 0 | 631 (94%) | 135 (93%) | 89 (95%) |
| 0 | 446 (66%) | 97 (67%) | 77 (82%) | 1 | 40 (5.9%) | 8 (5.5%) | 1 (1.1%) |
| 1 | 170 (25%) | 34 (23%) | 12 (13%) | 2 | 2 (0.3%) | 2 (1.4%) | 4 (4.3%) |
| 2 | 41 (6.1%) | 11 (7.6%) | 5 (5.3%) | Unknown | 7 |  |  |
| 3 | 19 (2.8%) | 3 (2.1%) |  | cv (Vascular fibrous) | | | |
| Unknown | 4 |  |  | 0 | 434 (64%) | 71 (49%) | 41 (44%) |
| t (Tubulitis) | | | | 1 | 158 (23%) | 43 (30%) | 22 (23%) |
| 0 | 558 (82%) | 107 (74%) | 50 (54%) | 2 | 51 (7.6%) | 21 (14%) | 17 (18%) |
| 1 | 95 (14%) | 29 (20%) | 18 (19%) | 3 | 31 (4.6%) | 10 (6.9%) | 14 (15%) |
| 2 | 20 (2.9%) | 5 (3.5%) | 13 (14%) | Unknown | 6 |  |  |
| 3 | 7 (1.0%) | 3 (2.1%) | 12 (13%) | ah (Arteriolar hyalinosis) | | | |
| Unknown |  | 1 | 1 | 0 | 438 (65%) | 82 (57%) | 46 (49%) |
| I (Interstitial inflammation) | | | | 1 | 136 (20%) | 34 (23%) | 27 (29%) |
| 0 | 590 (87%) | 125 (87%) | 56 (60%) | 2 | 47 (7.0%) | 18 (12%) | 12 (13%) |
| 1 | 72 (11%) | 14 (9.7%) | 13 (14%) | 3 | 53 (7.9%) | 11 (7.6%) | 9 (9.6%) |
| 2 | 16 (2.4%) | 5 (3.5%) | 13 (14%) | Unknown | 6 |  |  |
| 3 | 1 (0.1%) | 1 | 12 (13%) | Bx | | | |
| Unknown | 1 |  |  | D | 424 (62%) | 94 (65%) | 39 (41%) |
| ptc (Peritubular capillaritis) | | | | P | 162 (24%) | 27 (19%) | 46 (49%) |
| 0 | 501 (74%) | 98 (68%) | 71 (76%) | Z | 94 (14%) | 24 (17%) | 9 (10%) |
| 1 | 101 (15%) | 29 (20%) | 12 (13%) | Age | 45 (33, 55) | 47 (34, 57) | 52 (31, 61) |
| 2 | 59 (8.7%) | 16 (11%) | 10 (11%) | Unknown | 11 |  |  |
| 3 | 19 (2.8%) | 2 (1.4%) | 1 (1.1%) | Gender | | | |
|  |  |  |  | Unknown | 1 (0.1%) |  |  |
|  |  |  |  | male | 420 (62%) | 89 (61%) | 59 (63%) |
|  |  |  |  | female | 259 (38%) | 56 (39%) | 35 (37%) |
|  |  |  |  | 1 n (%); Median (IQR) |  |  |  |

Supplementary Table S1 Patient Clinicopathologic Characteristics after applying sampling criterion in training (n=680), internal (n=145) and external (n=94) test sets. Tubulitis – infiltration of renal tubules by mononuclear cells (t, from 0 to 3),), interstitial inflammation–- infiltration of interstitium by mononuclear cells (i, from 0 to 3), glomerulitis–- margination of inflammatory leukocytes in the glomerular capillary loops (g, from 0 to 3), transplant glomerulopathy - interposition of mesangium and thickening of GBM (cg, from 0 to 3), interstitial fibrosis–- interstitial structure replaced by fibrosis (ci, from 0 to 3), Peritubular capillaritis – margination of inflammatory cells in the peritubular capillaries(ptc, from 0 to 3), tubular atrophy – interstitial tubules involuted (ct, from 0 to 3), Arterial fibrointimal thickening – expansion of intima between endothelium and media (cv, from 0 to 3), mesangial matrix expansion - increase of thickness of mesangial matrix (mm, from 0 to 3), arteriolar hyalinosis – nodular deposition of hyaline (ah, from 0 to 3). D – The transplant kidney biopsy is done either to screen or to diagnose a malfunctioning kidney, P – protocol kidney biopsy (PKB) is performed at 3 months post-transplantation, Z – Zero-time kidney biopsy are obtained at time of transplantation.
