# Supplementary material for: Computational pathology model to assess acute and chronic transformations of the tubulointerstitial compartment in renal allograft biopsies
Source: Sci Rep. 2024 Mar 4;14:5345. doi: 10.1038/s41598-024-55936-3 (PMC10912734; doi:10.1038/s41598-024-55936-3)
Supplement: Supplementary file 4 — Supplementary Table S2. [file 41598_2024_55936_MOESM4_ESM.docx]

|  | rSC | rSCs | tLumen% | tCell% | iMatrix% | iColl% | ptc% | tSize | tCellDen | PC1 | PC2 |
| --- | --- | --- | --- | --- | --- | --- | --- | --- | --- | --- | --- |
| rSC | 1 |  |  |  |  |  |  |  |  |  |  |
| rSCs | -0.41775 | 1 |  |  |  |  |  |  |  |  |  |
| tLumen% | -0.35101 | 0.257792 | 1 |  |  |  |  |  |  |  |  |
| tCell% | 0.155561 | -0.02708 | -0.09398 | 1 |  |  |  |  |  |  |  |
| iMatrix% | -0.09392 | 0.015125 | -0.2904 | -0.74275 | 1 |  |  |  |  |  |  |
| iColl% | -0.00584 | -0.01776 | -0.1852 | -0.78619 | 0.414529 | 1 |  |  |  |  |  |
| ptc% | 0.251446 | -0.35813 | -0.03725 | -0.08898 | -0.27295 | 0.13968 | 1 |  |  |  |  |
| tSize | -0.09554 | 0.093729 | 0.218854 | -0.12301 | -0.20039 | 0.181344 | 0.345691 | 1 |  |  |  |
| tCellDen | 0.235278 | -0.15288 | -0.11706 | -0.09503 | 0.217095 | 0.142153 | -0.39288 | -0.60025 | 1 |  |  |
| PC1 | 0.073838 | -0.06358 | -0.37711 | -0.53295 | 0.696039 | 0.532006 | -0.39845 | -0.63196 | 0.722057 | 1 |  |
| PC2 | 0.407796 | -0.24444 | -0.7047 | 0.680545 | -0.43982 | -0.19869 | 0.081466 | -0.22344 | 0.138443 | -0.04095 | 1 |

Supplementary Table S2 Pairwise Correlation Coefficients among Renal Histopathological Parameters and Principal Components: This table displays the correlation statistics between various renal histopathological features, including reciprocal Serum Creatinine (rSC), reciprocal Serum Creatinine slope (rSCs) percentage of tubular lumen area (tLumen%), percentage of tubular cells area (tCell%), percentage of non-fibrillary matrix area (iMatrix%), percentage of fibrillary collagen area (iColl%), percentage of peritubular capillaries area (ptc%), mean area of tubules (tSize), densities of cells in tubules (tCellDen), and the first two principal components (PC1, PC2) derived from a principal component analysis (PCA) of the data.
