# Supplementary material for: Computational pathology model to assess acute and chronic transformations of the tubulointerstitial compartment in renal allograft biopsies
Source: Sci Rep. 2024 Mar 4;14:5345. doi: 10.1038/s41598-024-55936-3 (PMC10912734; doi:10.1038/s41598-024-55936-3)
Supplement: Supplementary file 5 — Supplementary Table S3. [file 41598_2024_55936_MOESM5_ESM.docx]

|  |  | Actual Value (user defined annotation) | |  |
| --- | --- | --- | --- | --- |
|  |  | Positive | Negative |  |
| Predicted Value (from AI network) | Positive | True Positive | False Positive |  |
|  | Negative | False Negative | True Negative |  |
| Definition | | Formula | | |
| • TP – True Positives – Correctly predicted pixel | |  | | |
| • TN – True Negatives – Correctly predicted pixel | |  | | |
| • FP – False Positives – Incorrectly predicted pixel | |  | | |
| • FN – False Negatives – Incorrectly predicted pixel | |  | | |
| • Precision – the ratio of correctly predicted positive pixels to the total predicted positive pixels. | | $Precision=\frac{\mathrm{TP}}{TP+FP}$ | | |
| • Recall – the ratio of correctly predicted positive pixels to all the pixels in the class. | | $Recall=\frac{\mathrm{TP}}{TP+FN}$ | | |
| • F1-Score – the weighted average of Precision and Recall. | | $F1-Score=2*\frac{Precision*Recall}{Precision+Recall}=\frac{\mathrm{TP}}{2*TP+FP+FN}$ | | |

*Supplementary Table S3A Definitions and Formulas for Segmentation Model Metrics. This table defines key performance indicators used in evaluating the segmentation model, including True Positives (TP), True Negatives (TN), False Positives (FP), and False*

| Variables | Precision | Recall | F1-Score |
| --- | --- | --- | --- |
| Tubules | 0.90664 | 0.978113 | 0.940065 |
| Capillaries | 0.703177 | 0.84246 | 0.766543 |
| Glomeruli | 0.96057 | 0.952561 | 0.956487 |
| Sclerotic Glomeruli | 0.920649 | 0.960807 | 0.939755 |
| Arteries | 0.81519 | 0.925308 | 0.857407 |
| Cortex | 0.91105 | 0.988987 | 0.948082 |
| Medulla | 0.954217 | 0.790836 | 0.855537 |

*Supplementary Table S3B Performance Metrics of Segmentation Model. This table presents the precision, recall, and F1-scores for each histopathological feature identified by the segmentation model, including Tubules, Capillaries, Glomeruli, Sclerotic Glomeruli, Arteries, Cortex, and Medulla.*
